# Mapping monthly rainfall erosivity in Europe

**DOI:** 10.1016/j.scitotenv.2016.11.123

**Published:** 2017-02-01

**Authors:** Cristiano Ballabio, Pasquale Borrelli, Jonathan Spinoni, Katrin Meusburger, Silas Michaelides, Santiago Beguería, Andreas Klik, Sašo Petan, Miloslav Janeček, Preben Olsen, Juha Aalto, Mónika Lakatos, Anna Rymszewicz, Alexandru Dumitrescu, Melita Perčec Tadić, Nazzareno Diodato, Julia Kostalova, Svetla Rousseva, Kazimierz Banasik, Christine Alewell, Panos Panagos

**Affiliations:** a European Commission, Joint Research Centre, Directorate D - Sustainable Resources, Via E. Fermi 2749, I-21027 Ispra (VA), Italy; b Environmental Geosciences, University of Basel, Bernoullistrasse 30, CH-4056 Basel, Switzerland; c The Cyprus Institute, 20 Konstantinou Kavafi Street, CY-2121 Nicosia, Cyprus; d Estación Experimental de Aula Dei, Consejo Superior de Investigaciones Científicas (EEAD-CSIC), 50009 Zaragoza, Spain; e Institute of Hydraulics and Rural Water Management, University of Natural Resources and Life Sciences, Muthgasse 18, AT-1190 Vienna, Austria; f Slovenian Environment Agency, Hydrology and State of Environment Office, Cesta 4. julija 67, SI-8270, Krško, Slovenia; g Faculty of Environmental Sciences, Czech University of Life Sciences Prague, Kamýcká 129, 165 21 Praha, 6 – Suchdol, Czech Republic; h Department of Agroecology, Aarhus University, Blichers Alle 20, 8830 Tjele, Denmark; i Finnish Meteorological Institute, P.O. Box 503, FI-00101 Helsinki, Finland; j Hungarian Meteorological Service, Budapest, Kitaibel Pál Street 1, HU-1024, Budapest, Hungary; k UCD Dooge Centre for Water Resources Research, University College Dublin, Ireland; l Department of Climatology, National Meteorological Administration, Bucuresti-Ploiesti 97, RO-013686, Romania; m Meteorological and Hydrological Service, Gric 3, HR-10000, Zagreb, Croatia; n Met European Research Observatory, 82100 Benevento, Italy; o Slovak Hydrometeorological Institute, Climatological service, Jeséniova 17, SK-83315 Bratislava, Slovakia; p Institute of Soil Science, Geotechnologies and Plant Protection, N. Poushkarov, Shosse Bankya Str. No7, BG-1336 Sofia, Bulgaria; q Warsaw University of Life Sciences, ul. Nowoursynowska 166,Warsaw PL-02-787, Poland

**Keywords:** REDES, R-factor, Seasonal rainfall intensity, Modelling, Soil erosion, K-means clustering, Cubist

## Abstract

Rainfall erosivity as a dynamic factor of soil loss by water erosion is modelled intra-annually for the first time at European scale. The development of Rainfall Erosivity Database at European Scale (REDES) and its 2015 update with the extension to monthly component allowed to develop monthly and seasonal R-factor maps and assess rainfall erosivity both spatially and temporally. During winter months, significant rainfall erosivity is present only in part of the Mediterranean countries. A sudden increase of erosivity occurs in major part of European Union (except Mediterranean basin, western part of Britain and Ireland) in May and the highest values are registered during summer months. Starting from September, R-factor has a decreasing trend. The mean rainfall erosivity in summer is almost 4 times higher (315 MJ mm ha^− 1^ h^− 1^) compared to winter (87 MJ mm ha^− 1^ h^− 1^).

The Cubist model has been selected among various statistical models to perform the spatial interpolation due to its excellent performance, ability to model non-linearity and interpretability. The monthly prediction is an order more difficult than the annual one as it is limited by the number of covariates and, for consistency, the sum of all months has to be close to annual erosivity. The performance of the Cubist models proved to be generally high, resulting in R^2^ values between 0.40 and 0.64 in cross-validation. The obtained months show an increasing trend of erosivity occurring from winter to summer starting from western to Eastern Europe. The maps also show a clear delineation of areas with different erosivity seasonal patterns, whose spatial outline was evidenced by cluster analysis. The monthly erosivity maps can be used to develop composite indicators that map both intra-annual variability and concentration of erosive events. Consequently, spatio-temporal mapping of rainfall erosivity permits to identify the months and the areas with highest risk of soil loss where conservation measures should be applied in different seasons of the year.

## Highlights

• Rainfall erosivity is mapped intra-annually for the first time at European scale.
• The modelling is based on a developed monthly Rainfall Erosivity Database at European Scale (REDES).
• REDES data is modelled with WorldClim covariates using Cubist regression trees.
• Using Cubist erosivitiy is effectively spatially estimated over Europe for each month.
• Seasonal patterns of erosivity are further analyzed using clustering techniques.

## Graphical abstract

**Figure:**
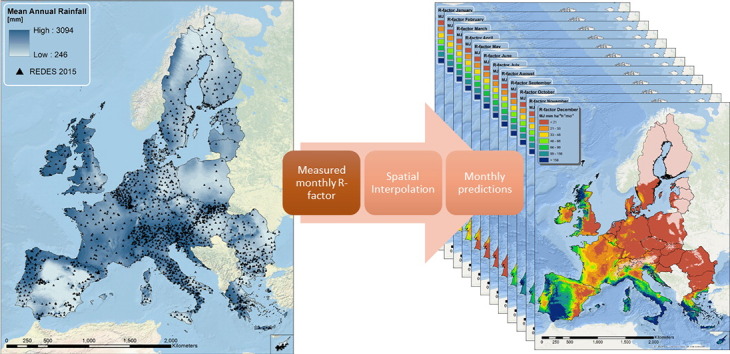

## 1 Introduction

Rainfall is essential for plant development, biomass and agriculture but it also is the driving force for water erosion processes through detachment of soil particles and formation of surface runoff (Nyssen et al., 2005). Soil erosion prediction is of crucial importance for appropriate land management and soil use (Oliveira et al., 2013). Soil erosion models play an important role in soil erosion predictions and among them the USLE (Wischmeier and Smith, 1978) and RUSLE (Renard et al., 1997) are the most widely used. Rainfall is the main driver for soil erosion by water and the relationship between rainfall and sediment yield is given by rainfall erosivity (Yang and Yu, 2015). Rainfall erosivity is calculated from a series of single storm events by multiplying the total storm kinetic energy with the measured maximum 30-minute rainfall intensity (Wischmeier and Smith, 1978).

Among the soil erosion risk factors rainfall erosivity and land cover/management are considered the most dynamic factors to change during the year. The rainfall erosivity variability affects agriculture, forestry, hydrology, water management, and ecosystem services. Consequently, neglecting the seasonal variability of rainfall erosivity and as a result the intra-annual soil loss variability, may lead to improper decision making (Wang et al., 2002). Rainfall erosivity shows different patterns among the wet and dry seasons both in terms of magnitude and in relationship to rainfall amount (named erosivity density) (Hoyos et al., 2005, Meusburger et al., 2012, Borrelli et al., 2016, Panagos et al., 2016a). Monthly erosivity has been studied in some regions in Europe such Portugal (Ferreira and Panagopoulos, 2014, Nunes et al., 2016), Sicily (D'Asaro et al., 2007) and Calabria (Terranova and Gariano, 2015) in Italy, Ebro Catchment in Spain (Angulo-Martínez and Beguería, 2009), western Slovenia (Ceglar et al., 2008), north-eastern Austria (Klik and Konecny, 2013) and Czech Republic (Janeček et al., 2013). Nevertheless an assessment of monthly erosivity over Europe is still missing.

The recent development of the Rainfall Erosivity Database at European Scale (REDES) and the annual rainfall erosivity map of Europe (Panagos et al., 2015a) is based on high temporal resolution rainfall data collected across all European Union countries and Switzerland. The main objective of this study is to capture the spatial and temporal variability of rainfall erosivity in the European Union and Switzerland based on high temporal resolution rainfall data. Specific objectives of this study are to:

- Model monthly and seasonal rainfall erosivity based on 1568 precipitation stations in all countries of the European Union and Switzerland
- Apply a spatial interpolation model which best maps R-factor spatial and temporal distribution
- Compare the produced seasonal maps of R-factor to the distribution of Köppen-Geiger climate maps
- Apply a cluster analysis to assess the patterns of rainfall erosivity across Europe
- Derive maps of the variability, density and seasonal peaks of rainfall erosivity

## 2 Data and methodology

### 2.1 Rainfall Erosivity Database at the European Scale (REDES)

**Fig. 1:**
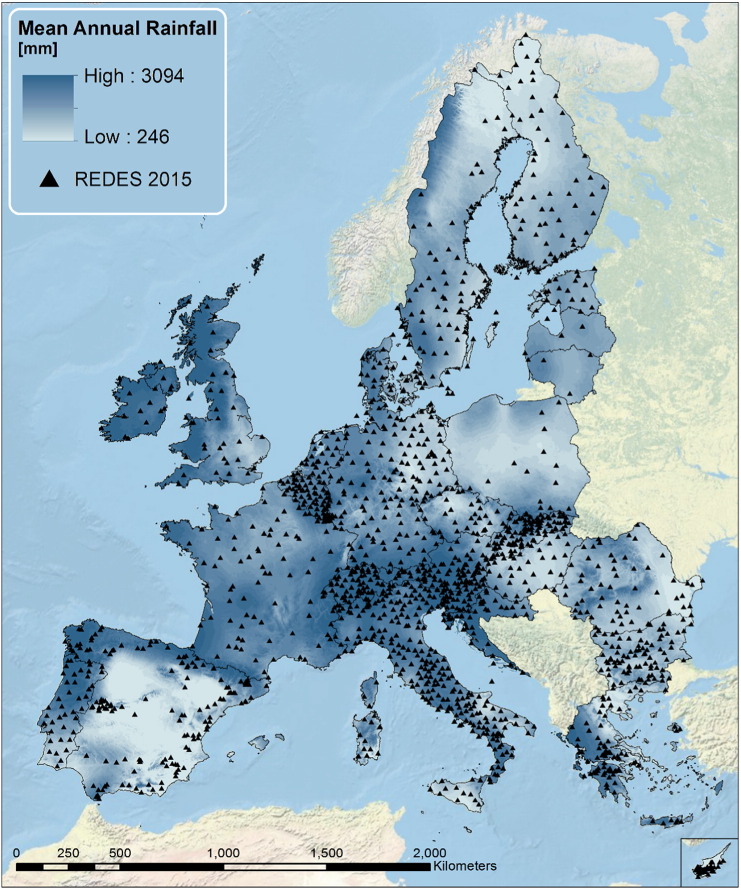
Rainfall stations included in the Rainfall Erosivity Database at European Scale (REDES).

The Rainfall Erosivity Database at the European Scale (REDES) has been developed from high temporal resolution rainfall data collected from 1568 stations from all European Union countries and Switzerland (Fig. 1). A participatory approach has been followed in the data collection as the high temporal resolution rainfall records have been collected from meteorological and environmental services from countries with the collaboration of scientists in the domain of rainfall erosivity (Panagos et al., 2015b). REDES includes > 29 × 10^3^ years of data with an average of 17.5 year-data per station. For further details on REDES, please see Panagos et al. (2015a).

Wischmeier (1959) proposed the “Rainfall Erosion Index” on a seasonal or annual basis as a product of storm energy and its maximum 30-minute intensity (EI). Unfortunately, very few datasets at European scale are available with breakpoint data (Porto, 2015). The available high temporal resolution rainfall data are at different time resolutions (5 min, 10 min, 15 min, 30 min and 60 min). As a first step, the rainfall erosivity has been calculated in the original resolution for each month and precipitation station. Then, it was decided to transform all the calculated monthly R-factors to a common resolution of 30 min. For this reasons, Panagos et al. (2016b) have developed monthly calibration factors at different time resolutions. Finally, 18,816 monthly R-factor values are serving for making the interpolations and developing the monthly R-factor maps.

### 2.2 Monthly rainfall erosivity calculation

Monthly rainfall erosivity (R-factor) is calculated for each of the 1568 stations as a long-term (17.5 years) monthly average of the erosivity values of a certain month. The monthly R-factor is an average value, calculated as a summation of EI_30_ for each month, divided by the number of observed years. According to Brown and Foster (1987), EI_30_ is defined as the product of the kinetic energy of rainfall events (E) and its maximum 30-minute intensity (I_30_).

(1) $$R_{j}=\frac{1}{n}\sum_{j=1}^{n}\sum_{k=1}^{\mathrm{mj}}\left({\mathrm{EI}}_{30}\right)k$$

where: R_j_ is the average monthly rainfall erosivity (MJ mm ha^− 1^ h^− 1^ mo^− 1^); n is the number of years recorded; m_j_ is the number of erosive events during a given month j; and EI_30_ is the rainfall erosivity index of a single event k. The erosivity EI_30_ (MJ mm ha^− 1^ h^− 1^) of a single event is defined as:

(2) $${\mathrm{EI}}_{30}=\left(\sum_{r=1}^{m}\mathrm{er}\mathrm{vr}\right)I_{30}$$

where e_r_ is the unit rainfall energy (MJ ha^− 1^ mm^− 1^) and v_r_ the rainfall volume (mm) during the r-th period of a storm which divided into m parts. I_30_ is the maximum 30-minute rainfall intensity (mm h^− 1^).

The unit rainfall energy (e_r_) is calculated for each time interval as follows (Brown and Foster, 1987):

(3) $$e_{r}=0.29\left[1-0.{72\;e}^{\left(-0.05\mathrm{ir}\right)}\right]$$

where i_r_ is the rainfall intensity during the time interval (mm h^− 1^).

The sums of EI_30_ and the average R-factor have been calculated on a monthly basis. The erosive events have been selected based on the three criteria of Renard et al. (1997) applied by Panagos et al. (2015a).

### 2.3 Support covariates and spatial model approach

As evidenced by Panagos et al. (2015b) the values of the R-factor are strongly correlated with monthly average precipitations and monthly average temperature extremes. The climatic variables of interest were obtained from the WorldClim (http://www.worldclim.org/) dataset similar to the previous study (Panagos et al., 2015a). WorldClim data layers are the interpolated values of average monthly climate data collected from numerous weather stations worldwide during the period 1950–2000. The WorldClim spatial data layers have been established by a thin plate smoothing spline with latitude, longitude and elevation as independent variables to locally interpolate the station data (Hijmans et al., 2005).

Though the WorldClim data are known to be subject to spatial biases in mountainous regions as the Alps, the Carpathians, and the Dinarides (Machac et al., 2011, Perčec Tadić, 2010), we decided to base our parameterization on such data for following reasons. Firstly, the WorldClim is a global database, thus it avoids the time-consuming process of cross-border harmonization when using regional or local databases such as the HISTALP (Auer et al., 2007) or the CARPATCLIM (Spinoni et al., 2015). Secondly, its higher spatial resolution (~ 1 km at mid-European latitudes) which is more suitable for spatial models including soil features that usually need even higher resolution (500 m). Other European datasets at lower resolution (e.g., the gridded E-OBS at 0.25°; Haylock et al., 2008) have been tested, but resulted in a high loss of information at local scale. Finally, we plan extending the rainfall erosivity climatology to future climatic scenarios. Consequently the availability of a downscaled set of climate simulations from the WorldClim data (see: http://www.worldclim.org/cmip5_30s) plays a key role in the choice of this database for this study.

Given the correlation between the R-factor and climatic data, a regression approach was used in order to infer the distribution of rainfall erosivity from WorldClim's covariates. The climatic variables utilized in the model were:

1. average monthly precipitation
2. average minimum & maximum monthly precipitation
3. average monthly temperature
4. bioclimatic variables

As all these layers derive directly from the WorldClim database, references about the methodology adopted to derive them and a description of the files can be found in Hijmans et al. (2005).

In order to estimate the R-factor for each month a regression model was built as:

(4) $${\mathrm{RF}}_{i}=\sum_{i}^{n}f_{j}\left({\mathrm{Tmi}}_{\;i\ldots n},{\mathrm{Tmx}}_{\;i\ldots n},{\mathrm{Pavg}}_{i\ldots n,},{\mathrm{Bio}}_{i\ldots m}\right)$$

where *RF*_*i*_ is the R-factor value for month *I*, *f*_*j*_ represent a set of functions (of arbitrary complexity) selected by the learning algorithm; *Tmi*_*i* … *n*_ , *Tmx*_*i* … *n*_ , *Pavg*_*i* … *n*,_ , *Bio*_*i* … *m*_, are a set of covariates corresponding layers 1–4 enumerated above. Since the relation between the R-factor and climatic variables is likely non-linear, a more flexible approach (compared to the linear regression) to regression has to be used. In this case regression coefficients are substituted by generic functions *f*_*j*_ whose additive combination develops the model. In the previous study by Panagos et al. (2015a), the R-factor was estimated by using Gaussian Process Regression (GPR) (Rasmussen and Williams, 2005) with radial basis kernels for non-linear mapping; although successfully in estimating R-factor values, the GPR has the disadvantage of being a black-box technique, meaning that identifying the influence of a given input covariate on the prediction is not straightforward. Therefore, to have a better insight into the model structure, we chose to work with a regression tree technique, namely Cubist (Quinlan, 1992). This choice is further motivated by Cubist's excellent performance, its ability to model non-linearity and its interpretability. In particular, Cubist can model non-linear relations by building a series of piecewise regression models linked to form a smoothed function. For these reasons Cubist has been used to estimate and map a variety of features, such as soil organic carbon (Somarathna et al., 2016, Viscarra Rossel et al., 2016, Akpa et al., 2016), soil erosion by wind (Borrelli et al., 2014), forest biomass (Blackard et al., 2008, Freeman and Moisen, 2007), soil properties (Gray et al., 2015, Padarian et al., 2015). The Cubist model is additive and each month's R-factor model is initially fitted using the full set of covariates. In order to avoid issues due to the collinearity among covariates a supervised feature selection using simulated annealing (Kirkpatrick et al., 1983) was then performed on the starting set of covariates; then the Cubist fitting algorithm was applied on the set of selected features. This procedure was repeated for each one of the months to be estimated. Since only the most informative variables are selected for each model, the fitted models usually comprise different sets of covariates.

### 2.4 Prediction of monthly erosivity by Cubist model

Cubist is a rule–based model, where a decision tree is grown with the terminal leaves containing linear regression models. These models use the predictors to split the tree branches into intermediate linear models using a splitting criterion. As splitting criteria the standard deviation of the class in a given branch is treated as a measure of the error at that node and each attribute at the same node is tested by estimating the expected reduction in error. The attribute that is chosen for splitting is the one maximizing the expected error reduction. The standard deviation reduction (SDR) which is calculated by Eq. (5) corresponds to the expected error reduction:

(5) $$\mathrm{SDR}=\sigma \left(T\right)-\sum \frac{\left|T_{i}\right|}{\left|T\right|}\sigma \left(T_{i}\right)$$

where *T*_*i*_ corresponds to the sets that result from splitting the node according to the chosen attribute. At the end of the tree, the linear regression models at the leaves predict continuous numeric attributes, their combination is analogous to a piecewise linear functions and combine the result in a non-linear function. The splitting process terminates when the standard deviation is only a small fraction less than the standard deviation of the original instance set or when a few instances remain. Prediction estimates are calculated using the linear regression model at the terminal node of the tree and predictions are “smoothed” by taking into account the prediction from the linear model in the previous node of the tree. The tree can be reduced to a set of rules, while redundant rules are eliminated via pruning and/or combined for simplification.

Cubist also adopts two meta-learning rules in order to improve model performance. One is a boosting–like scheme called “committees” (Quinlan, 1992) where iterative model trees are created in sequence and the final prediction is an average of the predictions from each model tree. The second is “instance based correction” where nearest–neighbours are used to adjust the predictions from the rule–based model. Instead of estimating a value from a single combination of covariates (i.e. a stack of pixels from the covariates), Cubist founds the n closest observations in features space and pools them by averaging these training set observation. The reasoning behind this procedure is that pooling results in better estimates, likely by limiting the influence of noise in the data (Quinlan, 1993).

Cross-validation was used for assessing the goodness-of-fit (GOF) of the Cubist model. Thus, N random samples containing 10% of the original dataset were taken with replacement and left for validation. At each N iteration the model was calibrated with the remaining data and GOF metrics were computed for the validation sample. The metrics used to evaluate model performance are the coefficient of determination (R^2^), the root mean squared error (RMSE), the normalized root mean squared error (NRMSE) and the Mean Error Bias (MBE). This procedure (calibration and cross-validation) was repeated independently for each month.

### 2.5 Delimitation of R-factor spatial patterns

The maps obtained by Cubist prediction were pooled and clustered in order to search for an optimal number of, relatively, homogenous (in terms of rainfall erosivity seasonal patterns) areas. This can be done by adopting a clustering algorithm in order to identify clusters sharing similar properties. A suitable technique is using k-means clustering. K-means clustering, in its range of slightly different implementations, is commonly applied in soil (Carré and McBratney, 2005, Odgers et al., 2011) and climate (Favre et al., 2016, Rau et al., 2016, Santos et al., 2016) sciences where grouping of multiple variables is needed.

K-means (MacQueen, 1967) is a centroid-based clustering, where clusters are represented by a central vector. The number of clusters is fixed to k and the algorithm finds the k cluster centres and assign the objects to the nearest cluster centre, such that the squared distances from the cluster are minimized. Since the solution to the clustering problem can only be numerically approximated, numerical methods reused. In order to avoid the algorithm to get stuck in a local minimum the best of multiple runs is selected, with the initial centres chosen randomly.

However the number of clusters k is arbitrarily chosen by the user. To optimize the number of clusters into which subdivide the data, we used the Calinski-Harabasz index (Calinski and Harabasz, 1974). This index expresses the ratio between the between-group scatter (BGSS) and the within-group scatter (WGSS); the first quantity expressing how far are clusters barycentre from whole data barycentre, the second expressing how far is each observation in a cluster from that cluster barycentre. The optimal number of clusters is then found by testing different possible number of clusters and selecting the combination with the highest index (the one that maximizes BGSS and minimizes WGSS).

## 3 Results and discussion

### 3.1 Fits of the Cubist regression model for individual months

**Fig. 2:**
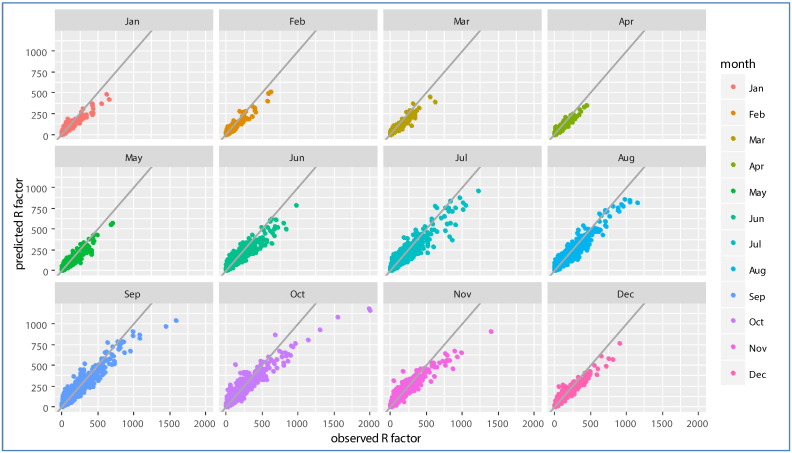
Observed vs predicted values of R-factor.

For each month, a different Cubist model was fitted. In general, the cross-validation approach showed that all the models performed quite well with performances, expressed in terms of R^2^, ranging between 0.46 and 0.61 (Table 1). The RMSE ranged between 34.19 and 115.45. The MBE is usually intended to indicate average model ‘bias’; that is, average over- or underestimation. MBE values are generally low for all the models excluding the one for the month of June which shows a more relevant underestimation, this is also evident from Fig. 2 where a large part of the points falls below the bisector line.

It should be noted that spring months' R-factor values are generally less well predicted than winter and summer months' in terms of R^2^(Table 1). However, in terms of NRMSE, summer months have the largest error; this is due to the higher variability of the R-factor. It is quite clear that summer (and autumn) months tend to have a wider range and higher variability of R-factor values, which partly accounts for the higher error (Fig. 2). However, from the MBE it is evident that for some months, the model is biased when predicting high values of rainfall erosivity. This behaviour is evident in Fig. 2, where values of R-factor above 700–800 MJ mm ha^− 1^ h^− 1^ mo^− 1^ are constantly underestimated by the model. This is probably due to the occurrence of extremely intensive events whose distribution cannot be captured by a model using monthly averaged covariates. Nevertheless, given the scarce number of extreme events with R-factor > 1000 MJ mm ha^− 1^ h^− 1^ in REDES (99.9% of the events are below 989.97 MJ mm ha^− 1^ h^− 1^) we can trust model predictions for what matters the main seasonal and spatial trend of R-factor.

In general, each month R-factor is predicted by a different set of covariates automatically selected by the Cubist model. The resulting residuals of the Cubist regression showed no spatial correlation, so a subsequent kriging of the residuals was not done.

### 3.2 Monthly R-factor predicted maps

**Fig. 3:**
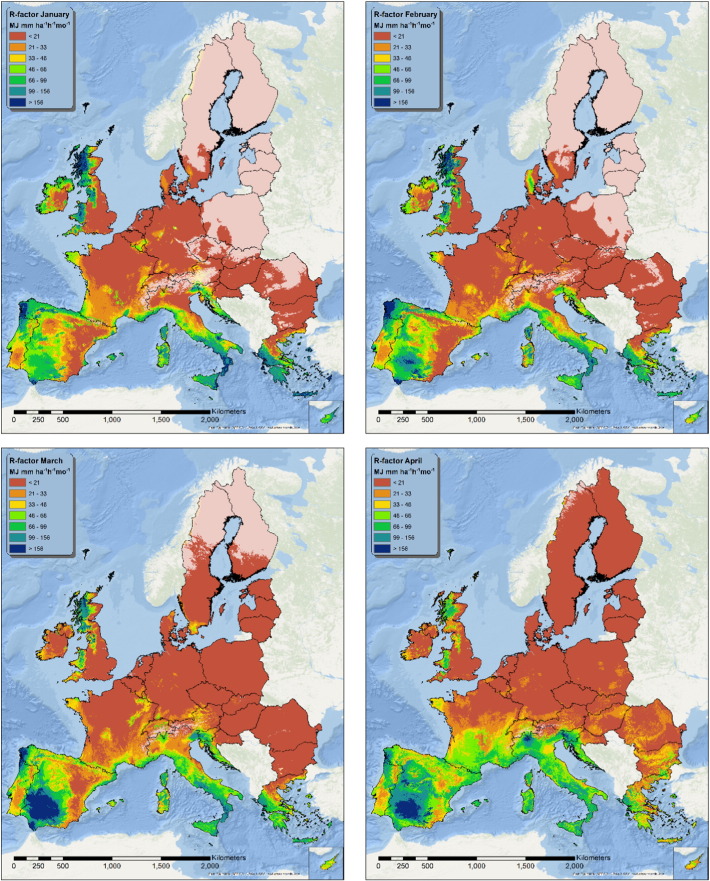
Maps of estimated R-factor from January to April.

**Fig. 4:**
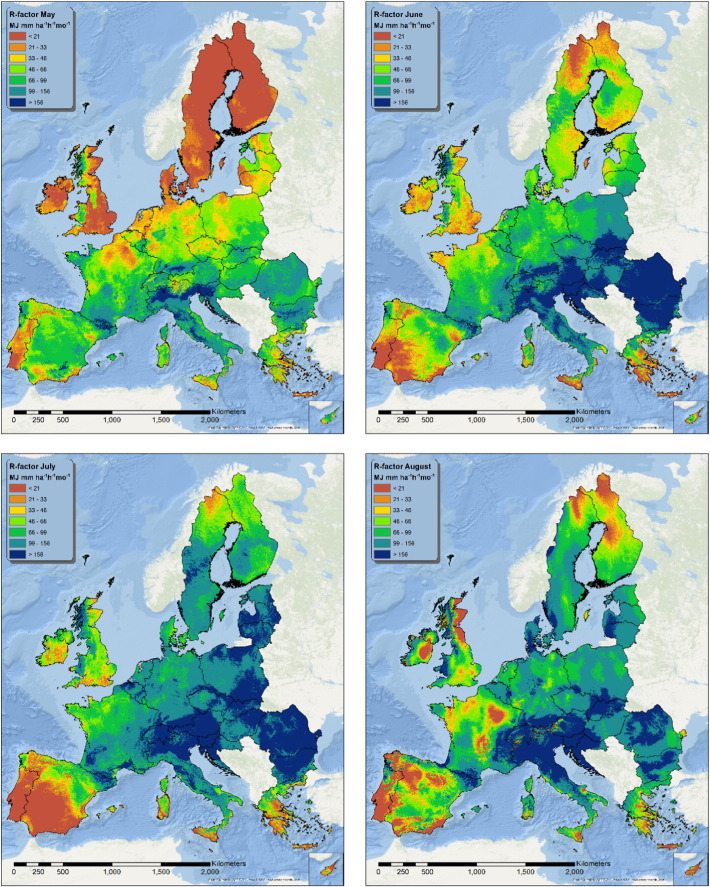
Maps of estimated R-factor from May to August.

**Fig. 5:**
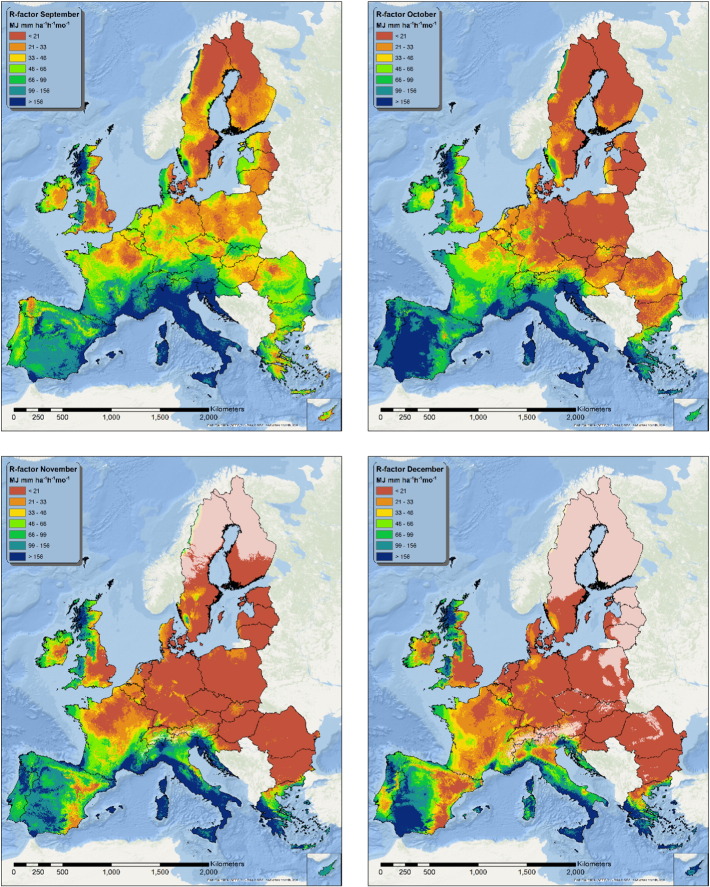
Maps of estimated R-factor from September to December.

The twelve fitted models were applied on the spatially exhaustive set of covariates in order to produce monthly maps of the estimated R-factor (Fig. 3, Fig. 4, Fig. 5). During winter, part (or the totality) of the precipitation is constituted by snow; for this reason, areas where the average maximum monthly temperature is below 0°C are whitened in the maps. In these areas the model still predicts a value for R-factor, but the predictions are likely to be unrealistic.

The pattern of the R-factor follows the typical seasonality of precipitations that characterizes different European climatic zones. The Mediterranean area shows high rainfall erosivity values from September to January, while the area surrounding the Alps, the Carpathians and the Balkans shows its maximum erosivity from June to August.

The northern Atlantic coast, Ireland, Wales and part of Scotland show higher summer rainfall erosivity values, although the intensity in this areas is lower than in the Mediterranean or the Alpine region. Eastern Europe and Sweden follow the same seasonal pattern, but with generally lower values of rainfall erosivity.

Remarkably, the sum of monthly R-factor values, with a small margin of approximation, corresponds well to the yearly estimates presented by Panagos et al. (2015a). By calculating the difference between the two maps the resulting difference has a mean value of 15.3 MJ mm ha^− 1^ h^− 1^ and a standard deviation of 159 MJ mm ha^− 1^ h^− 1^. These values imply that the estimates of the R-factor derived by the cumulative sum are, on average, 2% higher than those obtained by the direct estimation of the yearly R-factor. This difference is still within the 0.95 confidence interval for the difference of the means (as calculated by a Welch Two Sample *t*-test, Welch, 1947) of both the yearly estimates and the monthly estimates sum of the R-factor.

While cross-validation ensures that the models are properly fitted, it cannot provide an assessment of the prediction where observations are not present.

### 3.3 Seasonal R-factor

**Fig. 6:**
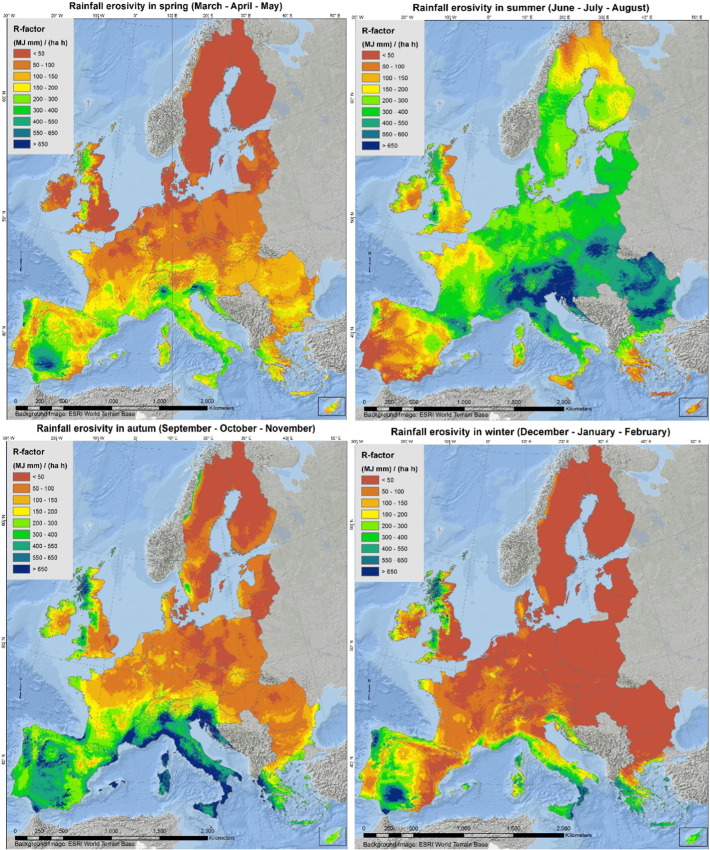
Rainfall erosivity (MJ mm ha^− 1^ h^− 1^) per season (winter – spring – summer –autumn).

In the European Union and Switzerland, the mean rainfall erosivity in summer is 315 MJ mm ha^− 1^ h^− 1^ which is almost 4 times higher than in winter (87 MJ mm ha^− 1^ h^− 1^). Due to higher values in the Mediterranean basin, the mean rainfall erosivity in autumn is 203 MJ mm ha^− 1^ h^− 1^ compared to the 116 MJ mm ha^− 1^ h^− 1^ in spring. With the exception of the Mediterranean basin, the general spatial patterns of rainfall erosivity both in seasonal maps (Fig. 6) and monthly maps (Fig. 3, Fig. 4, Fig. 5) exhibit a smooth increase of R-factor from winter to spring, followed by a sharp intensification in summer and then a smooth decrease in autumn. The highest divergence is noticed in autumn (followed by winter) with low mean values in Central and Northern Europe and very high values in the southern part. The objective of the seasonal R-factor maps (Fig. 6) is to show the seasonal patterns in European Union. We recognize that seasons are not the same around Europe, but we preferred a simplified approach based on aggregation of data on 3-month basis. Due to availability of data, end-users may do their customized seasonal maps in any region of European Union.

### 3.4 Seasonal erosivity by Köppen-Geiger climate classification and cluster analysis

**Fig. 7:**
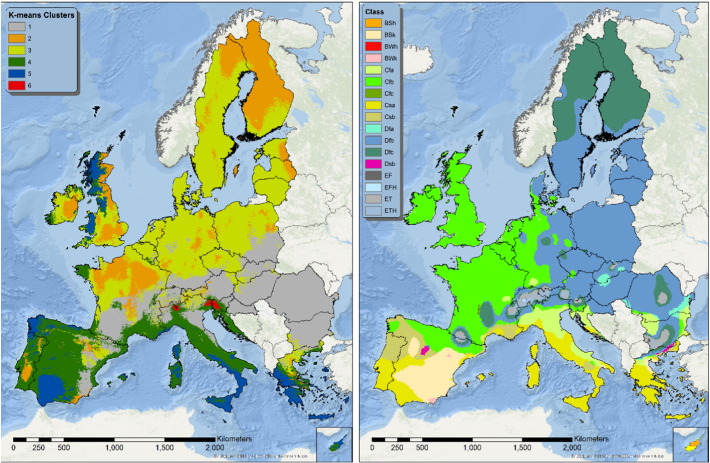
Spatial distribution of R-factor clusters (left) compared to Köppen-Geiger climate zones (right).

The map of Köppen-Geiger climate classification is commonly used by researchers to evaluate the output of climate modelling (Peel et al., 2007); an updated map is available at a 0.1° resolution by Peel et al. (2007). The inspiration of Köppen classification stem from vegetation mapping and as such the classification is based on more parameters than simple precipitation intensity. A different approach is to use the R-factor values itself to identify relatively homogeneous areas in terms of rainfall erosivity seasonal patterns by cluster analysis. In this study the best split according to the Calinski-Harabasz index is given by a split in six clusters. Fig. 7 depicts the spatial distribution of the clusters (on the left) and Köppen-Geiger climatic zones (right). At a first glance, it is evident that clusters' distribution does not follow the Köppen-Geiger climate subdivision. This in an interesting result as Köppen-Geiger classes are often used to discriminate between different climatic zones, but in the case of rainfall erosivity they appear to be a non-optimal choice. Nevertheless, while accounting for the different resolution of the two maps, some of the subdivisions are remarkably similar. The demarcation between eastern and western Italy and Greece and the north-south subdivision of Portugal. However the transition between Temperate and Continental climates appears to be placed further East in the Köppen map. Moreover clustering shows a marked division along the south-west to north-east direction that is present, but not so evident in the Köppen map.

**Fig. 8:**
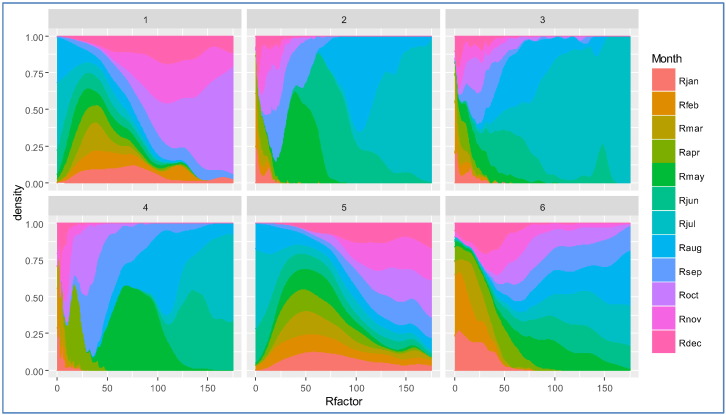
Monthly R-factor contribution in each cluster. The horizontal axis expresses the value of the R-factor in MJ mm ha^− 1^ h^**−** 1^.

Nevertheless clusters capture most of the variability of the R-factor in Europe. The fact that different clusters represent quite different precipitation regimes can be evidenced by plotting values of R-factor densities by month and cluster. Fig. 8 shows how much each month contributes to different values of R-factor per cluster. Each box represents a different cluster, while the coloured area represents each month's contribution to a given level of R-factor. So, as an example, for cluster 3 the totality of events with an R-factor between 150 and 175 MJ mm h^− 1^ ha^− 1^ occurs in July.

Cluster 1 represents areas with a prevalence of highly erosive events in autumn (Figs. 8). Clusters 2 and 3 have a prevalence of events in late spring and early summer, with cluster 2 experiencing more events in summer. Cluster 4 presents important contribution of late spring and early autumn months. Cluster 5 shows a prevalence of high R-factor values in autumn and a still significant contribution of winter months. Cluster 6 is peculiar for its limited spatial extension, however, it shows very high values of R-factor during summer months that are quite uncommon in other clusters.

**Fig. 9:**
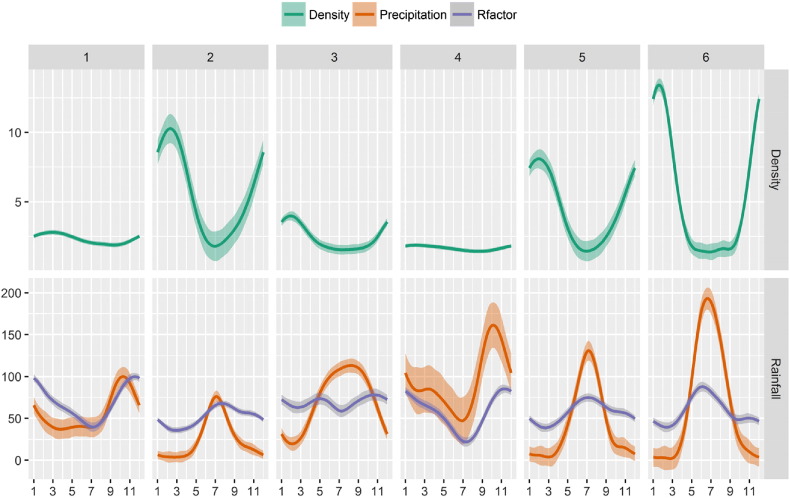
Monthly R-factor, Precipitation and Erosivity Density contribution in each cluster. The horizontal axis is month of the year. The shaded regions represent the 0.95 confidence intervals.

**Fig. 10:**
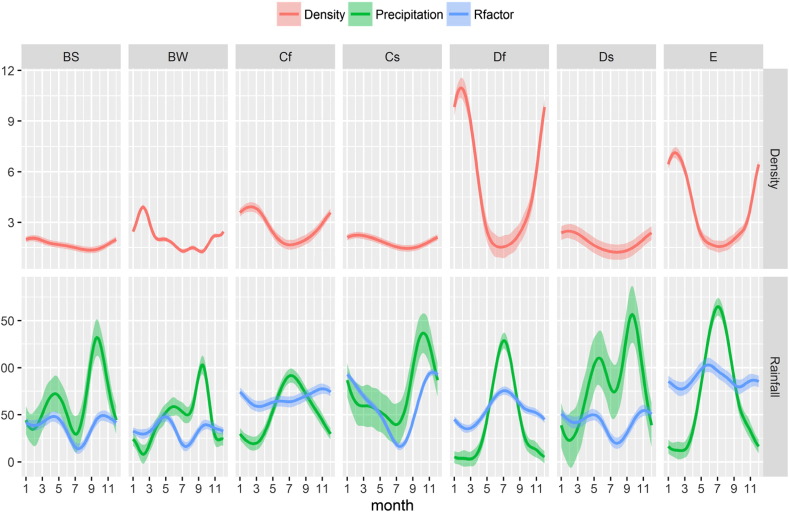
Monthly R-factor, Precipitation and Erosivity Density by Köppen-Geiger main climatic zones (BS: Steppe, BW: Desert; Cf: Temperate without dry season; Cs: Temperate, dry summer; Df: Cold without dry season; Ds: Cold, dry summer; E: Polar). The shaded regions represent the 0.95 confidence intervals.

Another comparison between Köppen climate zones and clusters can be made by plotting the seasonal trend for each cluster/class. Fig. 9, Fig. 10 show R-factor and precipitation trends grouped by cluster/class. Monthly Erosivity Density (MED) was added to the plots for comparison. MED is obtained by diving the monthly erosivity by average monthly rainfall (Bonilla and Vidal, 2011, Kinnell, 2010). Regions and seasons of high erosivity density indicate a higher risk of erosive rainstorms and, as a consequence, high erosion and flooding (Dabney et al., 2011, Panagos et al., 2016a). Clusters 2, 5 and 6 are characterised by high erosivity density during winter months as are climate zones Df (Cold without dry season) and E (Polar). However, in the map, class E corresponds only to high altitude areas and is probably subject to estimation issues.

### 3.5 Ratio of the least erosive month to the most erosive

The estimation of R values for the twelve months of the year allows the production of several indicator maps, something that would not be possible with just yearly estimates. The monthly maps of Fig. 3, Fig. 4, Fig. 5 depict a quite large difference in erosivity. Across Europe, a comparable difference is present in the temporal dimension where the same area can have values of R orders of magnitude dissimilar in different times of the year. While the maps of Fig. 3, Fig. 4, Fig. 5 give a general idea about the areas with the highest variability in rainfall erosivity across the year, a better understanding can be obtained by creating a map of the ratio between the lowest and the highest erosivity values. The ratio value can be calculated as

(6) $$\varphi_{j}=\frac{\min\left(X_{i=1,\ldots ,12,j}\right)+1}{\max\left(X_{i=1,\ldots ,12,j}\right)+1}\;$$

Where *j* denotes a specific pixel in each of the twelve *i* monthly rasters and *X* denotes the values of rainfall erosivity. The constant value of 1 is added to avoid values of *φ*_*j*_ tending to 0 when the lowest values of *X* tends to zero.

**Fig. 11:**
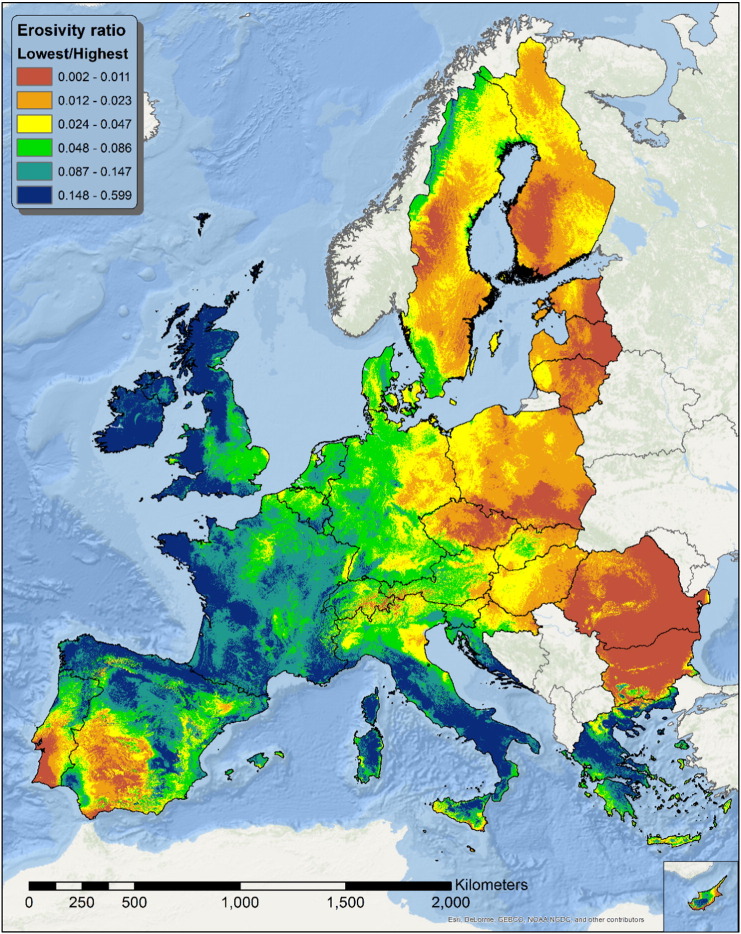
Ratio between the least and the most erosive month R-factor.

The resulting map is shown in Fig. 11, where values close to 0 represent areas where the difference in R between the most and the least erosive months is larger. Fig. 11 also shows an East-West demarcation analogue to the Köppen map, with lower ratios in North-Eastern Europe, South Portugal and Western Andalusia.

### 3.6 Weighted erosivity density

The annual erosivity gives information on the total rainfall energy, but provides no information about the time distribution of the events. Moreover it tells nothing about the concentration of extreme events during the year. The same annual erosivity can result from numerous events with little relative energy or from few very energetic events; obviously with a different outcome.

An assessment of the distribution of erosivity over the year is critical for management and mitigation procedures, so the development of composite indicators expressing not only the yearly estimate, but also the relative impact of extreme events is critical for soil conservation.

In this study we attempted to develop a composite indicator summarizing the intra-annual variability as well as its dependence by extreme events. This indicator is expressed as the ratio between annual erosivity density and the Coefficient of Variation (CV) of monthly erosivity density.

The CV is expressed as the ratio between the standard deviation (σ) and the mean (μ) and shows the extent of variability in relation to the mean. In the context of rainfall erosivity density the *CV*_*MED*_ was calculated as:

(7) $${\mathrm{CV}}_{\mathrm{MED}}=\frac{\sigma_{\mathrm{MED}}}{\mu_{\mathrm{MED}}}\;\text{where}:\;\mu_{\mathrm{MED}}=\frac{\sum_{i=1}^{12}{\mathrm{MED}}_{i}}{12};\sigma_{\mathrm{MED}}=\sqrt{\frac{\sum_{i=1}^{12}{\left({\mathrm{MED}}_{i}-\mu_{\mathrm{MED}}\right)}^{2}}{12}}$$

**Fig. 12:**
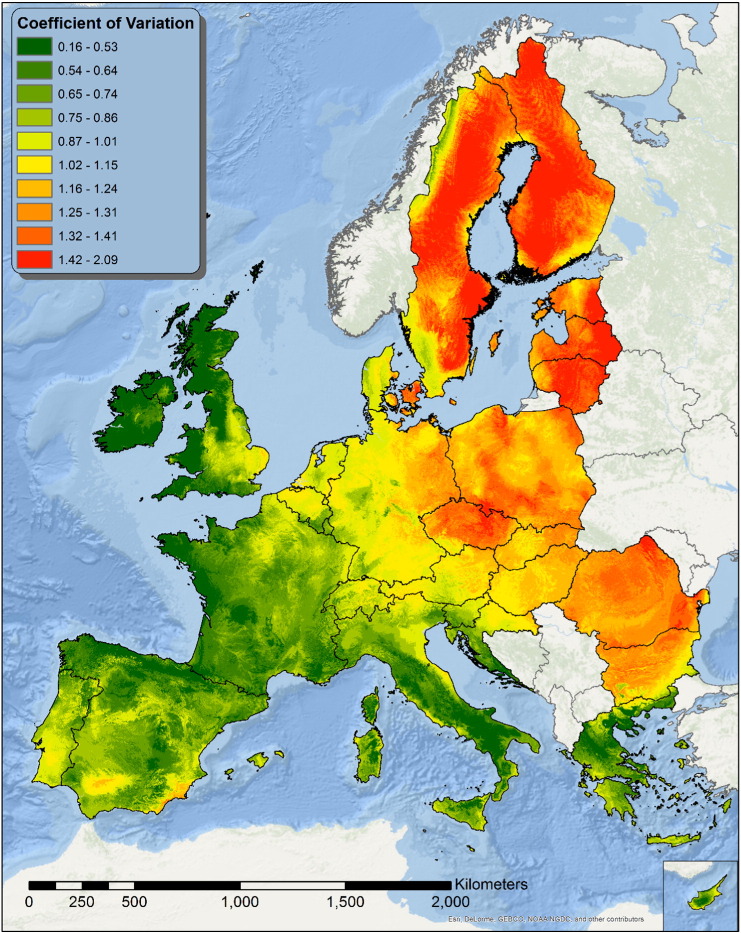
Map of the Coefficient of Variation of the Monthly Erosivity Density. Areas with values < 1 are subject to more evenly distributed events, while areas where CV > 1 are subject to a more heterogeneous precipitation regime during the year.

**Fig. 13:**
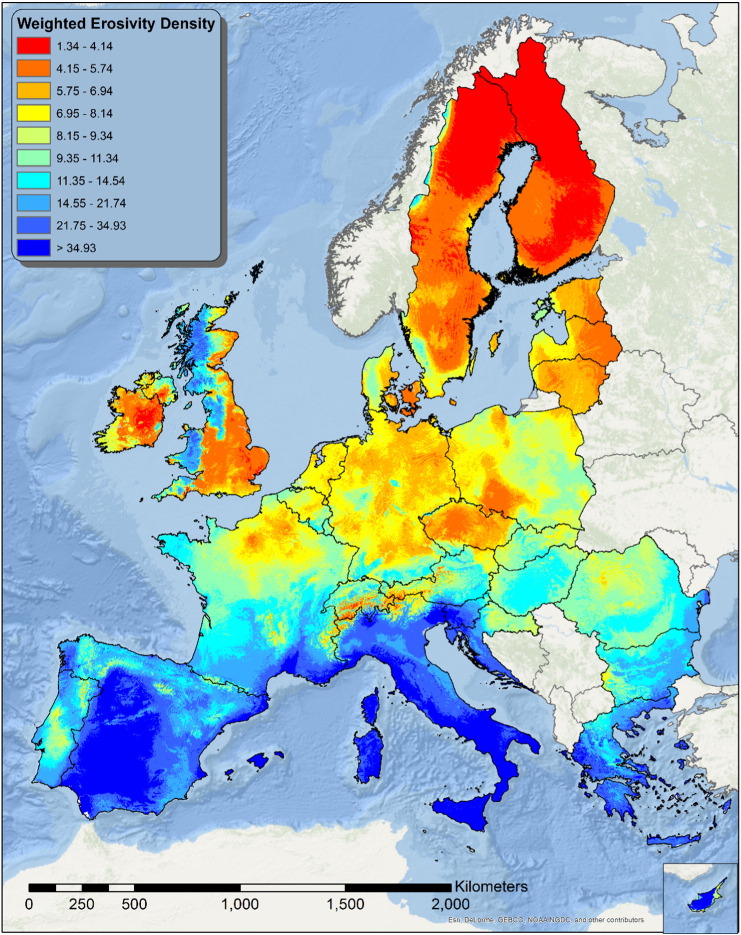
Weighted Erosivity Density (WED). Areas with the highest WED are more subject to extreme erosive events.

Where *MED*_*i*_ is the value of estimated monthly erosivity density for month *i*. The value of *CV*_*MED*_ is maximised when the monthly values of MED differ a lot over the year (*σ*_*MED*_ ≫ *μ*_*MED*_) and minimized when the values are more or less evenly distributed (*σ*_*MED*_ ≪ *μ*_*MED*_) (Fig.12). Thus, multiplying the annual Erosivity Density value by *CV*_*MED*_ it is possible to obtain a map of the Weighted Erosivity Density (WED) showing the areas where extremely energetic events are more likely to occur (Fig. 13).

Compared to the map of R-factor map by Panagos et al. (2015a), the map of WED (Fig.13) shows a different distribution with higher values in central Spain, Sicily and Sardinia and lower values along the Atlantic coast (Galicia, Bay of Biscay, Western Scotland, and Wales) and in parts Northern Italy. The map of the WED is an advancement of the annual erosivity density map (Panagos et al., 2015a) as it incorporates the monthly variation. This is particularly important for area where intense events have a potentially dramatic impact on soil erosion (Martınez-Casasnovas et al., 2002).

### 3.7 Months with highest and lowest erosivity

**Fig. 14:**
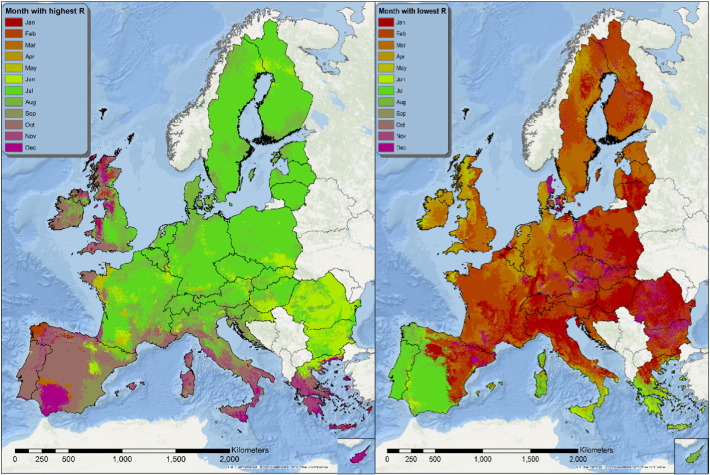
Map of the month of the year with the highest value of R (left) and lowest (right).

Another essential information about rainfall erosivity is the time of the year when erosivity is at its maximum as well as when it is at its minimum. The monthly estimation of the R-factor allows the mapping of month of the year corresponding to the most erosive one (Fig. 14). The same intensity of rainfall erosivity will result in a different effect on the soil erosion according to the time of the year when it occurs. This depends on different factors, like crop cover, that change during the year. Knowledge about the time of the year when the highest erosivity occurs is then critical for management practices, as it allows optimize mitigation procedures. For example in areas where the maximum erosivity occurs between September and October leaving crop residues on the ground as erosion protection would be recommended.

## 4 Data availability, limitations and comparability with local studies

The monthly erosivity datasets (GeoTIFF format) at 500 m resolution are available for free download in the European Soil Data Centre (ESDAC): http://esdac.jrc.ec.europa.eu, while the calculated erosivity values per station in REDES will become available in the future based on the agreed copyright issues with data providers.

The monthly erosivity datasets produced in this study should not be seen as challenging any local or regional erosivity maps. Local erosivity maps using local data of better quality (higher resolution or longer time-series) are of course more accurate. It is auspicable that local data and knowledge could be used to further improve the REDES database in the future.

The seasonal R-factor maps were qualitatively compared with regional studies in Italy, Spain, Portugal, Austria, Slovenia and Czech Republic. In the Ebro Catchment (Spain), Angulo-Martínez and Beguería (2009) have modelled the highest erosivity during November, October, September and May which is very similar to our monthly R-factor maps (Fig. 4, Fig. 5, Fig. 14). In this catchment, the lowest erosivity is estimated in summer and winter months. In Calabria (Italy), similar to the maps of Terranova and Gariano (2015), we predicted higher erosivity in January compared to March while October and November are the most erosive months and August the least erosive (Fig. 14). In Sicily (Italy), D'Asaro et al. (2007) estimated the summer erosivity to be equal to or slightly higher than the winter one while we noticed the highest values during September and October. In Algrave region, our results show similar patterns to de Santos Loureiro and de Azevedo Coutinho (2001) who have modelled the highest erosivity in October, November and December and the lowest in summer months. In Southern part of Portugal, similar to Nunes et al. (2016), we have estimated the highest erosivity the 3-months October–December followed by erosivity the erosivity during January–March while the lowest erosivity is during summer. Similar to our results, In South-eastern Portugal (Alqueva Dam Watershed) Ferreira and Panagopoulos (2014) have modelled the highest erosivity during autumn (c.a. 50%) and the lowest in summer while winter and spring have similar patterns. In the northeaster part of Austria, Klik and Konecny (2013) calculated the rainfall erosivity most dominant during the period May to July (60% of the total) followed by the period August to October (37%) which are very similar to our spatial patters (Fig. 4, Fig. 5). Comparing our results to the local study of Ceglar et al. (2008) in western Slovenia, the most erosive period is August to September (Fig. 14). In Czech Republic, Janeček et al. (2013) estimated that 85% of erosivity is taking place during summer which is close to our estimate (Fig. 6).

## 5 Conclusions

The spatial models' prediction of monthly European R-factors was satisfactory in terms of R^2^ and RMSE. Spring months are in general less well predicted than the rest of the year. However, the largest error is noticed in summer months due to higher variability of the R-factor. The predictions of R-factor monthly values over 800 MJ mm ha^− 1^ h^− 1^ are underestimated by the model, however, there are very few observations with values that are so high.

The intra-annual variability of rainfall erosivity is very high in Europe with July having a mean value of 115.1 MJ mm ha^− 1^ h^− 1^ which is almost 5 times higher than the mean value of January. Summer is the period with the highest R-factor and it is remarkable that around 55% of total rainfall erosivity in Europe takes place within only 4 months (June–September). However, the intra-annual distribution of erosivity and the concentration of extreme events have a high spatial variability in Europe. The clustering of erosivity in Europe (by k-means) showed that Köppen-Geiger climate classification is always not optimal for representing the spatio-temporal patterns of rainfall erosivity. Cluster analysis outlines a different and more complex spatial pattern of rainfall erosivity.

In this context, the monthly erosivity maps allowed the development of indicators for studying the intra-annual variability of erosivity and the concentration of erosive events. The variation of monthly erosivity and the ratio between the lowest/highest erosive month showed that Ireland, UK, west France, North west Spain, central south Italy and Greece have much lower intra-annual erosivity variation, compared to Eastern Europe and Scandinavia. The weighted monthly erosivity density allows to identify higher concentration of erosive events in southern Italy and central Spain compared to the Atlantic coast. Finally, the identification of the most erosive month allows to recommend certain agricultural management practices (crop residues, reduced tillage) in regions with high erosivity.

The spatio-temporal rainfall erosivity analysis at European scale is a first step towards developing dynamic (monthly, seasonal) maps of soil loss by water erosion. Besides soil erosion mapping, the intra-annual analysis of rainfall erosivity is an important step towards flood prevention, hazard mitigation, ecosystem services, land use change and agricultural production.

## Conflict of interest

The authors confirm and sign that there is no conflict of interest with networks, organisations and data centres referred to in this paper.

## Figures and Tables

**Table 1:** Cubist model cross-validation performances for monthly R-factor interpolation (R^2^: coefficient of determination, RMSE: Root Mean Squared Error, NRMSE: Normalized Root Mean Squared Error, MBE: Mean Bias Error).

	R^2^	RMSE	NRMSE	MBE
Jan	0.498	42.07	0.064	− 0.60
Feb	0.504	38.09	0.061	− 1.71
Mar	0.508	36.12	0.058	− 0.21
Apr	0.473	34.19	0.077	− 0.82
May	0.462	53.03	0.075	2.76
Jun	0.494	79.82	0.082	15.14
Jul	0.519	92.66	0.076	0.92
Aug	0.590	87.51	0.076	3.05
Sep	0.613	97.20	0.061	7.52
Oct	0.475	115.45	0.058	0.45
Nov	0.536	91.61	0.065	− 2.86
Dec	0.607	59.72	0.066	− 1.23
